# Early Endothelial Progenitor Cells (eEPCs) in systemic sclerosis (SSc) - dynamics of cellular regeneration and mesenchymal transdifferentiation

**DOI:** 10.1186/s12891-016-1197-2

**Published:** 2016-08-12

**Authors:** S. Patschan, D. Tampe, C. Müller, C. Seitz, C. Herink, G. A. Müller, E. Zeisberg, M. Zeisberg, E. Henze, D. Patschan

**Affiliations:** 1 Clinic of Nephrology and Rheumatology, University Hospital of Göttingen, Robert-Koch-Straße 40, 37075 Göttingen, Germany; 2 Clinic of Dermatology, University Hospital of Göttingen, Robert-Koch-Straße 40, 37075 Göttingen, Germany

## Abstract

**Background:**

Patients with systemic sclerosis (SSc) are endagered by tissue fibrosis and by microvasculopathy, with the latter caused by endothelial cell expansion/proliferation. SSc-associated fibrosis potentially results from mesenchymal transdifferentiation of endothelial cells. Early Endothelial Progenitor Cells (eEPCs) act proangiogenic under diverse conditions. Aim of the study was to analyze eEPC regeneration and mesenchymal transdifferentiation in patients with limited and diffuse SSs (lSSc and dSSc).

**Methods:**

Patients with both, lSSc and dSSc were included into the study. The following parameters were evaluated: eEPC numbers and regeneration, concentrations of vasomodulatory mediators, mesenchymal properties of blood-derived eEPC. Serum samples of healthy subjects and SS patients were used for stimulation of cultured human eEPC, subsequently followed by analysis of mesenchymal cell characteristics and mobility.

**Results:**

Twenty-nine patients were included into the study. Regenerative activity of blood-derived eEPCs did not differ between Controls and patients. Circulating eEPC were significantly lower in all patients with SSc, and in limited and diffuse SSc (lSSc/dSSc). Serum concentrations of promesenchymal TGF-b was elevated in all patients with SSc. Cultured mononuclear cells from SS patients displayed higher abundances of CD31 and of CD31 and aSMA combined. Finally, serum from SSc patients inhibited migration of cultured eEPCs and the cells showed lower sensitivity towards the endothelin antagonist Bosentan.

**Conclusions:**

The eEPC system, which represents an essential element of the endogenous vascular repair machinery is affected in SSc. The increased appearance of mesenchymal properties in eEPC may indicate that alterations of the cells potentially contribute to the accumulation of connective tissue and to vascular malfunction.

**Electronic supplementary material:**

The online version of this article (doi:10.1186/s12891-016-1197-2) contains supplementary material, which is available to authorized users.

## Background

Systemic Sclerosis (SSc) is characterized by severe microvasculopathy, causing ongoing hypoperfusion of skin and inner organs [1]. Pathological analysis reveals endothelial cell proliferation in small blood vessels, subsequently leading to vascular obstruction (‘onion skin lesions’) [2]. Another hallmark in SSc is the accumulation of collagen fibers in skin, lungs, heart, and intestine. Such fibrosis or sclerosis can dramatically affect the functional integrity of organs/the whole organism [3]. The etiopathogenesis of the disease is far from being understood and although therapeutic measures are often intended to modulate the immune response in order to inhibit vasculopathy and fibrogenesis, the prognosis of SSc patients is quite poor since the course of the disease remains unaffected by drug therapy in many cases [4]. Thus, it can at least be doubted whether the cellular and molecular processes, responsible for endothelial cell proliferation and collagen accumulation are exclusively autoimmune by nature.

The field of vascular biology has significantly been emerged in recent years. This is particularly the case with regard to vascular repair mechanisms. In 1997, Asahara and colleagues described a population of blood-derived cells, critically involved in neovascularization [5]. These cells, termed Endothelial Progenitor Cells (EPCs) are heterogenous in terms of origin and phenotype [6–8]. They can promote post-ischemic vascular regeneration by both, direct and indirect mechanisms. Early Endothelial Progenitor Cells (eEPC) represent one major subpopulation of EPCs and they have meanwhile been used for therapeutic purposes in different experimental situations and in humans suffering from ischemic diseases [9–13]. Several recent investigations evaluated numbers of circulating eEPC in SSc [14–16]. However, in none of the studies, regenerative activity of eEPC was analyzed in SSc. We therefore aimed to quantify eEPC regeneration in SSc and utilized a Colony-Forming Unit Assay as described and reviewed previously [11, 17–19]. Our particular interest focused on phenotypic characteristics, possibly associated with a pro-mesenchymal switch of the cells within the (peri)vascular microenvironment.

## Methods

### Design and patients

The present investigation was a prospective single-center analysis. All patients were treated at the department of nephrology and rheumatology (University Hospital of Göttingen, Germany) between 2011 and 2013. All included individuals were classified according to the 2013 ACR(American College of Rheumatology)/EULAR(European League Against Rheumatism) criteria [20]. Differentiation between limited and diffuse SSc was made in accordance with the criteria published by LeRoy and Medsger [21]. The term ‘diffuse (generalized) disease’ is intended to describe patients with skin involvement, extended even to proximal areas of arms and legs including possible manifestations at the trunk (thorax and abdomen). These patients suffer more often and, if present, in many cases from more serious organ involvement. However, patients with limited disease may also show organic manifestations such as interstitial lung disease and esophageal damage. Lung involvement was defined as a lower than normal diffusion capacity (below 80 % of the nominal value) and/or as interstitial lung affection in radiographic analysis (chest x-ray or CT (Computed Tomography) scan) and/or as pulmonary hypertension. The latter was diagnosed by transthoracic echocardiogram. Esophageal manifestation was defined as either persistent dysphagea and/or as pathological esophagram. Joint affection was defined clinically if patients suffered from arthralgia. Renal involvement was defined by either increased serum creatinine and/or by proteinuria of above 150 mg/day. The patients underwent variable treatment regimens including administration of immunosuppressants for different reasons. For further clinical characterization a number of different parameters, such as c-reactive protein and different autoantibodies including anti-Scl70 and anti-Centromer were measured and collected in the database of the Clinic of Nephrology & Rheumatology (University Hospital of Göttingen, Germany). The control subjects (*n* = 16) matched the patients in terms of gender and age. They also signed written consent. Individuals in the control group were recruited from the staff of the University Hospital of Göttingen.

### eEPC quantification and regeneration

Quantification of peripheral circulating eEPC and analysis of eEPC regeneration was performed as described in numerous previous studies [11, 13, 18, 19, 22]. Nevertheless, in order to supply a better understanding of the parameters used for detecting eEPCs from the blood and for evaluating eEPC regeneration, the methodical approaches shall be outlined more in detail. Quantification of peripheral circulating eEPCs was performed by flow cytometry. Mononuclear cells (MNCs) were isolated by density gradient centrifugation using Histopaque-1077 solution (Sigma Diagnostics, St. Louis, MO) from ≈ 7.5 ml of heparinized peripheral blood. Cells were initially incubated for 1 h (on ice) using the following antibodies: rabbit anti CD133 (ab16518 – Abcam, Cambridge, UK), mouse anti human VEGFR2 (Vacular Endothelial Growth Factor Receptor 2 - KDR, directly conjugated - FAB 3571 F – R&D Systems, Minneapolis, MN, USA), followed by secondary incubation with PE-conjugated goat anti rabbit Fab (VEGFR, 111-116-144 – Jackson Immunoresearch, Baltimore, PA, USA) for 30 min on ice, respectively. After incubation, cells were washed with PBS-BSA 1 % (w/v). Data were acquired using a FACScalibur cytometer (Becton Dickinson, Heidelberg, Germany) equipped with a 488 nm argon laser and a 635 nm red diode laser and analyzed using CellQuest software (Becton Dickinson, San Jose, CA, USA). The setup of FACScalibur was performed according to the manufacturer’s instructions using unstained and single-antibody stained cells. Specificity of staining was controlled by incubation with isotype-matched immunoglobulins. To quantify eEPCs, the numbers of CD133/KDR double-positive cells within the myelomonocytic cell population were counted. eEPC regeneration on the other hand was evaluated by a Colony-Forming Units (CFU) assay. The assay was performed by using the EndoCult Liquid Medium Kit® (StemCell Technologies, Vancouver, BC, Canada) per the manufacturer’s protocol. MNCs were resuspended in complete EndoCult medium and seeded at 5 × 10^6^ cells/well on fibronectin-coated tissue culture plates (BD Biosciences, Rockville, MD, USA). After 48 h, wells were washed with media and nonadherent cells were collected. Nonadherent cells were plated in their existing media at 10^6^ cells/well in 24 well fibronectin-coated tissue culture plates for three days. Only colonies with at least 20 cells, containing rounded cells in the middle and elongated cells at the periphery, were considered as CFU-EC (Colony Forming Unit-Endothelial Cell) colonies. The numbers of colonies (colonies/well) appearing after this period were counted. At least two members of the laboratory staff evaluated the numbers. They were blinded for the diagnosis and status of the investigated patients/controls. The phenotype of cells within the colonies was determined more in detail. For this purpose, cells were characterized by the uptake of DiI-labeled acetylated low density lipoprotein (Dil-acLDL) (Invitrogen, Carlsbad, CA, USA) and binding of FITC-labeled UE lectin (Sigma Diagnostics, St. Louis, MO). Cells were first incubated with 10 μg/ml DiI-ac-LDL at 37 °C for 1 h and later fixed with 2 % formaldehyde for 10 min, followed by incubation with UE(Ulex Europaeus) lectin at 37 °C for 1 h. In some experiments, cells were additionally stained for FSP-1 (see below). The number and of Dil-acLDL+/UE lectin +/ FSP-1+cells was counted by laser scanning microscopy using an inverted fluorescence microscope IX-71 (Olympus Deutschland GmbH, Hamburg, Germany) equipped with the appropriate excitation and emission filters (AHF Analysentechnik, Tübingen, Germany). In order to provide some information about concistency of the CFU-EC assay, we compared the cumulative mean colony numbers aquired from healthy controls evaluated in three different studies from the past [19, 22]. One of the three studies has not been published yet. The mean number of colonies in all studies was 29.9 ± 2.2. It did not significantly differ from the current mean in healthy controls (*p* = 0.15).

### Mesenchymal transdifferentiation analysis

For mesenchymal transdifferentiation analysis mononuclear cells, isolated by density gradient centrifugation using Histopaque-1077 solution (Sigma Diagnostics, St. Louis, MO) were seeded on fibronectin-coated dishes in EGM-2 medium (Endothelial cell medium-2, Clonetics, Lonza, Walkersville, MD, USA) at 5 × 10^6^ cells/well. After two days, non-adherent cells were removed and adherent cells were cultured for five further days in EGM-2. Cells were washed with PBS/BSA 1 % and primary incubation was performed with the following antibodies: Mouse Anti-Human CD31 (DAKO, M0823), Rabbit Anti-Human aSMA (alpha Smooth Muscle Actin) (Abcam, ab32575), or Rabbit Anti-Human FSP-1 (Fibroblast Specific Protein-1) (DAKO, A5114). Incubation was done for 12 h at 4 °C overnight in a humidified chamber. After washing the cells once with PBS/BSA 1 %, secondary incubation was performed for 1 h at room temperature using the following antibodies: Alexa Fluor 488 donkey anti-mouse or Alexa Fluor 555 donkey anti-rabbit. After additional washing steps cells were coated with DAPI containing Vectashield Mounting Medium (Sigma-Aldrich, Taufkirchen, Germany). The percentages of CD31+, and of CD31+/aSMA+, cells were counted by laser scanning microscopy using the same microscope as described above (inverted fluorescence microscope IX-71 - Olympus Deutschland GmbH, Hamburg, Germany). Results were given as percentages of double-positive cells per nuclei.

### Elisa studies

Measurements of serum Angiopoietin-1 and −2, VEGF, TGF-beta (Transforming Growth Factor-beta), and PDGF (Platelet Derived Growth Factor) were performed using commercially available kits (all from R&D, Wiesbaden, Germany) according to the manufacturer’s protocol.

### Serum stimulation of cultured eEPC and eEPC migration assay

For serum stimulation and cell migration experiments, a commercially available human EPC cell line was employed (Human CD133+ Endothelial Progenitor Cells, BioChain, CA, USA). Cells were seeded after the 4th passage on gelatine-coated 24-well plates. The migration assay was performed as described previously (18). In detail, an artificial wound was created after the cells completely covered the dish in a homogenous manner. A standardized stamp was used for wound induction, allowing to create comparable wound areas. Shortly before wound induction, the incubation procedure was initiated. Serum samples from either healthy subjects or from SSc patients were diluted in EGM-2 (serum:medium-ratio = 1:5 or 1:7.5) and 600 μl were administered to the cells for 8 h in total. In some experiments Bosentan was additionally applied at 2000 or at 1000 ng/ml for the same period. At the beginning and 5 h after the end of the incubation procedure, wound areas were quantified. For analysis of EndoMT, cells were directly grown on glass bottom slides which allowed a more detailed microscopic investigation. The cell treatment protocol was the same. The staining procedure for endothelial (CD31) and mesenchymal markers (aSMA) and the subsequent analyses were performed as described earlier (see *Mesenchymal transdifferentiation analysis*).

### Statistical analysis

The results were expressed as mean ± SEM. The means of two populations were compared by Student’s *t* test. Three or more groups were compared by two-way ANOVA. Differences were considered significant at *p* < 0.05.

## Results

### Patients

A total number of 29 patients with systemic sclerosis was included into the study. The limited form was diagnosed in 21 (female: 19, male: 2), the diffuse type in eight patients (female: six, male: two). The mean age of all patients was 56 ± 2.5 years with 58 ± 2.5 years in patients with limited and 50 ± 5.7 years in patients with generalized SS. The mean duration of the disease was 4.5 ± 0.8 years in all patients with 4.6 ± 0.9 years in limited and 4.2 ± 1.5 years in diffuse SSc. The respective autoantibody profiles are shown in Additional file 1: Table S1. Pulmonary involvement was diagnosed in one patient with lSSc (4.7 %) and in two patients with dSSc (25 %). Other organ manifestations were: esophagus - lSSc 28 % (*n* = 6), dSSc 37.5 % (*n* = 3), joints - lSSc 47.6 % (*n* = 10), dSSc 50 % (*n* = 4), kidney - lSSc 9.5 % (*n* = 2), dSSc 0 % (*n* = 0). Fourteen patients with lSSc (66 %) and three patients with dSSc (37.5 %) suffered from (systemic) arterial hypertension. Digital ulcers were diagnosed in three patients with lSSc (14.2 %) and in two subjects (25 %) with dSSc. At the time of inclusion into the study immunomodulatory/immunosuppressive treatment was performed in five patients (cyclophosphamide 1, prednisolone only 2, prednisolone + azathioprine 1, interferone 1) with limited and in three patients (azathioprine 1, cyclophosphamide 1, cyclosporine A 1) with generalized disease. One patient with dSSc (12.5 %) received Bosentan for treating pulmonary hypertension. Additional file 1: Table S1 summarizes the clinical characteristics.

### eEPC numbers and regeneration

**Fig. 1:**
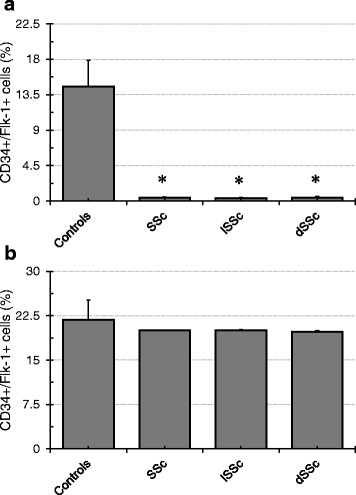
Percentages of peripheral circulating eEPC and eEPC regeneration in SSc. **a** shows CD133+/Flk-1+ cells (eEOCs). Percentages of double-positive cells significantly differed between Controls and all SSc categories (SSc all, lSSc, and dSSc) with lower values in SSc patients, respectively. **b** shows eEPC colony formation. Colony Forming Unit-Endothelial Cells (CFU-Ecs) were not different between the categories (Data as mean ± SEM, ✻ : *p* < 0.05 - for exact *p*-values see text)

Total numbers of circulating eEPCs (CD133+/Flk-1+ cells) significantly differed between controls and SSc patients: Controls 14.6 ± 2.9 %; SSc all 0.4 ± 0.1 %; lSSc ±0.4 0.1 %; dSSc 0.5 ± 0.3 %; the respective *p*-values were: Controls vs. SSc all *p* < 0.0001; Controls vs. lSSc *p* < 0.0001; Controls vs. dSSc *p* = 0.04 (Fig. 1). As opposed to previously published studies in certain rheumatic diseases (RA (Rheumatoid Arthritis), GPA (Granulomatosis with Polyangitis) (11, 22)) eEPC colony numbers were not different between the groups: Controls 21.8 ± 4.1; SSc all 20 ± 3; lSSc 20 ± 3.6; dSSc 19.8 ± 5.6; the respective *p*-values were: Controls vs. SSc all *p* = 0.7; Controls vs. lSSc *p* = 0.7; Controls vs. dSSc *p* = 0.8 (Fig. 1).

### Mesenchymal transdifferentiation of blood-derived eEPC

**Fig. 2:**
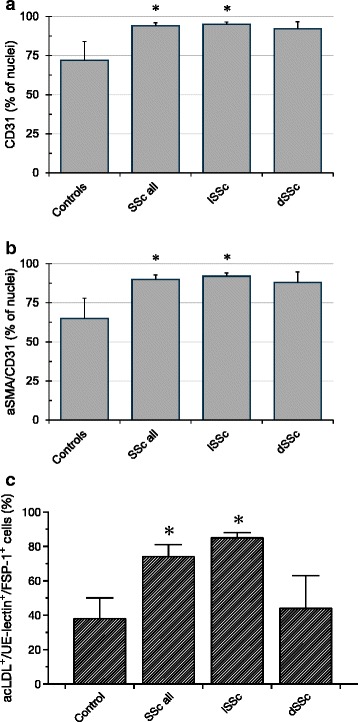
Mesenchymal transdifferentiation of blood-derived eEPCs in SSc. Expression CD31 was significantly higher in all patients with SSc and in lSSc, dSSc did not differ from Controls (**a**). **b** displays aSMA in CD31+ cells with higher percentages in SSc all and in lSSc. **c** shows results of additional marker staining (FSP-1), the results were comparable to those aquired with aSMA (Data as mean ± SEM, ✻ : *p* < 0.05 - for exact *p*-values see text)

Since SSc is characterized by collagen deposition in various types of tissues/organs, we aimed to investigate if phenotypic alterations of blood-derived eEPC possibly accompany the process of localized/generalized fibrosis. Our particular interest focused on the de-novo expression of mesenchymal marker proteins by the cells. Thus, mononuclear blood cells, cultured according to an established protocol for eEPC expansion were stained for CD31 and alpha-Smooth Muscle Antigen (aSMA). Patients with SSc (SSc all and lSSc) showed significantly higher percentages of CD31+ cells as compared to healthy controls whereas dSSc patients did not: Controls 72 ± 12 %; SSc all 94 ± 1.9 %; lSSc 95 ± 1.3 %; dSSc 92 ± 4.6 %; the *p*-values were: Controls vs. SSc all *p* = 0.01; Controls vs. lSSc *p* = 0.03; Controls vs. dSSc *p* = 0.18 (Fig. 2). In addition, percentages of CD31+ cells expressing aSMA were also higher in both, SSc all and lSSc: Controls 65 ± 13 %; SSc all 90 ± 2.8 %; lSSc 92 ± 2.1 %; dSSc 88 ± 6.7 %; the *p*-values were: Controls vs. SSc all *p* = 0.01; Controls vs. lSSc *p* = 0.02; Controls vs. dSSc *p* = 0.17 (Fig. 2).

These results were further confirmed by an additional analysis, using the mesenchymal marker FSP-1 (23). Cultured mononuclear cells were stained for UE lectin, acLDL, and FSP-1 and percentages of triple-positive cells were significantly elevated in all patients with SSc (SSc all) and in patients with limited disease (lSSc) (SSc all 74 ± 7 % and lSSc 85 ± 3 % vs. controls 38 ± 2 %, *p* = 0.01 and *p* < 0.0005 - Fig. 2).

### Angiomodulatory/profibrotic mediators

Serum concentrations of distinct vasomodulatory and pro-mesenchymal mediators were evaluated since impaired eEPC regeneration has been shown to be associated with defects in the Ang-1/-2 system. In addition, Ang-1/-2 and VEGF are known agonists of eEPCs under certain circumstances and TGF-beta has been identified as potent inductor of fibrogenesis in renal and other diseases [23, 24]. Concentrations of Angiopoietin-1 and −2, Platelet Derived Growth Factor (PDGF), and Vascular Endothelial Growth Factor (VEGF) did not differ between controls and either all patients with SSc, or with lSSc/dSSc (Ang-1 in pg/ml: Controls 2595 ± 609; SSc all 3309 ± 484; lSSc 2958 ± 418; dSSc 4478 ± 1530; all *p*-values >0.05; Ang-2 in pg/ml: Controls 1506 ± 162; SSc all 1877 ± 374; lSSc 1805 ± 429; dSSc 2117 ± 760; all *p*-values >0.05; VEGF in pg/ml: Controls 35 ± 10.5; SSc all 58 ± 12.8; lSSc 55 ± 16; dSSc 67.5 ± 14.3; all *p*-values >0.05; PDGF in pg/ml: Controls 48.8 ± 13; SSc all 77.7 ± 10.5; lSSc 67.5 ± 9.6; dSSc 111.7 ± 28.7; all *p*-values >0.05).

**Fig. 3:**
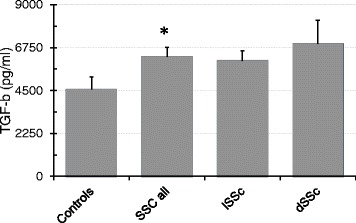
Pro-mesenchymal TGF-b in SSc. All Patients with SSc (SSc all) showed higher concentrations of TGF-b as compared to healthy controls (Data as mean ± SEM, ✻: *p* < 0.05 - for exact *p*-values see text)

Transforming Growth Factor-beta (TGF-b) was significantly higher in all patients with SSc as compared to the Controls (TGF-b in pg/ml: Controls 4549 ± 677; SSc all 6259 ± 495; lSSc 6057 ± 533; dSSc 6935 ± 1246; *p*-value for Controls vs. SSc all 0.049) (Fig. 3).

### Serum-induced eEPC migration and mesenchymal transdifferentiation

**Fig. 4:**
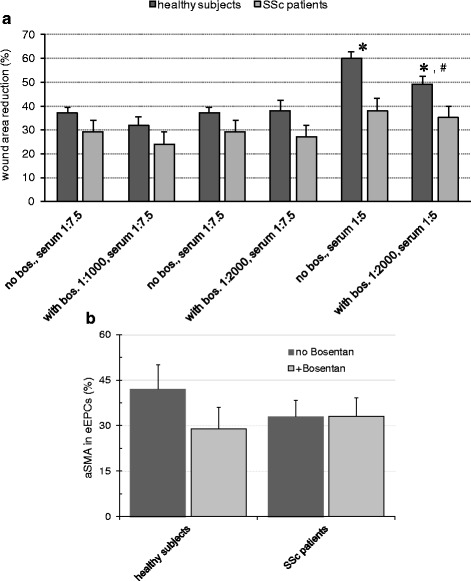
eEPC migration and EndoMT in vitro. Cultured human eEPCs were treated with serum samples from healthy controls, and from patients with SSc with versus without simultaneous administration of Bosentan to the culture medium. Analysis were performed at 5 h after beginning of the incubation. In general, migration of eEPC treated with patient serum was significantly slower (**a**). Bosentan reduced cellular migration in the ‘healthy subjects’ category. This effect exclusively occurred under the following experimental conditions: serum:medium-ratio 1:5, Bosentan concentration 2000 ng/ml. Expression of aSMA by the cells was comparable in all categories with no significant alteration by Bosentan (**b** – experimental conditions: serum:medium-ratio 1:5, Bosentan concentration 2000 ng/ml) (bos.: Bosentan; ‘serum’ represents the serum:medium-ratio; Data as mean ± SEM, ✻: differences between healthy subjects and SSc patients within a group significant with *p* < 0.05; #: differences between healthy subjects between two groups significant with with *p* < 0.05)

Finally, serum samples from healthy subjects and SSc patients were used for incubating cultured human eEPCs, followed by analysis of cell migration and mesenchymal transdifferentiation. Five hours after wound induction migration of eEPC, treated with serum from SSc patients was significantly slower as compared to those incubated with serum from healthy subjects (wound area reduction in %: healthy subjects 60 ± 2.9; SSc patients 38 ± 1.7; *p* < 0.0001). Simultaneous Bosentan administration to the culture medium reduced migration in the ‘healthy subject’ but not in the ‘SSc’ category (wound area reduction in %: healthy subjects 49 ± 3.5; SSc patients 35 ± 2.5; p for healthy subjects with versus without Bosentan administration 0.03). These effects exclusively occurred under the following conditions: serum:medium-ratio 1:5, Bosentan concentration 2000 ng/ml. Two further experimental settings were evaluated: serum:medium-ratio 1:7.5 with Bosentan 2000 ng/ml or 1000 ng/ml. However, although cells treated with serum from healthy controls tended to migrate faster in all groups, the differences as compared to SSc patients were not statistically significant. Figure 4 summarizes the results. Analysis of mesenchymal transdifferentiation of the cells revealed comparable levels of aSMA expression by the cells in healthy subjects and SSc patients with or without Bosentan treatment (eEPCs stained positive for aSMA in %: healthy subjects – no Bosentan 42 ± 8 and 33 ± 5 with Bosentan; SSc patients, no Bosentan 29 ± 7 and 33 ± 6 with Bosentan – Fig. 4). The differences were not statistically significant between any of the respective categories.

## Discussion

The two fundamental results of the current study were (I) the lower percentages of circulating eEPC all analyzed categories (SSc all, lSSc, dSSc) and (II) the pro-mesenchymal switch of cultured blood-derived eEPCs in SSc in general and in lSSc in particular. Another interesting finding was related to reduced migratory activity of cultured eEPCs in the presence of serum from SSc patients

The life-threatening nature of systemic sclerosis results from two pathological phenomenona: limited or generalized fibrosis and vascular obstruction/occlusion [25]. The latter is critically mediated by endothelial cell proliferation with subsequent intimal hyperplasia [26]. The mechanisms responsible for endothelial expansion have been studied in recent years but the current knowledge is still very limited. Among the factors that have been suggested to contribute to endothelial proliferation/functional transformation were chemical substances, vasculotropic viral pathogens, anti-endothelial cell autoantibodies, and others [2, 26–32]. In this context it has to be recognized that the endothelium does not only increasingly proliferate but also (most likely) undergoes a process of mesenchymal transdifferentiation or Endothelial-to-Mesenchymal Transition (EndoMT) [25]. In other situations, EndoMT has been documented to perpetuate cardiac and renal fibrosis [33–35]. SSc-related fibrogenesis significantly results from interstitial accumulation of myofibroblasts and these cells are being discussed to, at least in part originate from the vascular endothelium [25]. The microenvironmental humoral milieu is substantially affected in SSc. In a more recent study Guiducci and colleagues showed SSc-derived Mesenchymal Stem Cells (MSCs) to release numerous bioactive/proangiogenic factors that potentially stimulate angiogenesis and fibrosis (SDF-1, VEGF, TGF-beta) [36]. Such alterations, which are partly reflected by increases of circulating pro-mesenchymal substances (TGF-b - see results) may promote endothelial transdifferentiation into cells with mesenchymal properties. Several studies showed a critical role for Transforming Growth Factor-beta in mediating EndoMT [23, 35]. Early Endothelial Progenitor Cells have been documented to stabilize the microvasculature mostly by indirect mechanisms including the release of vasomodulatory mediators in close proximity to the endothelium [17]. Thus, eEPCs maintain the integrity of blood vessels under both, physiological and pathological conditions. The increased expression of mesenchymal proteins by the cells in SSc (SSc all and lSSc) has two possible implications. Firstly, it potentially reflects the inability of the cells to stabilize the endothelial ‘identity’ in a way that EndoMT ensues. Secondly, it is not a hallmark of functional eEPC incompetence but an attendant phenomenon of the complex humoral and cellular events that occur during the process leading to SS-associated fibrosis and vasculopathy. Regarding EndoMT, it can only be speculated whether dSSc patients did not show significantly higher percentages of CD31+/aSMA+ and acLDL+/BS-1+/FSP-1+ cells as compared to the Controls. In general, dSSc is associated with more severe end organ damage including more aggravated fibrosis and often higher risk and prevalence of digital ulcers. It somehow surprises that in comparison to controls, the two mentioned cell populations were not higher in dSSc. However, in the latter group immunosuppressive treatment was performed in 37.5 % as opposed to 23.3 % in the lSSc group. Low-dose chemotherapy for instance has been shown to reduce EPC mobilization into the blood of cancer patients [37]. Thus, the lack of differences between controls and dSSC may potentially also result from therapy-associated effects.

Another interesting result of our study was related to circulating eEPCs and to eEPC regeneration. CFU-ECs have been evaluated in several own studies, with altered colony formation in all investigations published so far [11, 13, 18, 19, 22]. The current study did not reveal impaired eEPC regeneration but a significant lack of circulating cells in all categories. One may argue that SSc is characterized by increased eEPC turnover, due to generalized microvascular damage and repair. In this respect, the normal colony numbers may reflect stimulated regeneration as well which nevertheless is not sufficient to compensate peripheral cell degradation. Nevertheless, to draw any definite conclusions is surely too early yet. At this point it has to be realized that other studies related to EPCs in SSc showed results, seemingly conflicting with our data. Allanore and colleagues found increased levels of CD34+/CD133+ cells in SSc as compared to osteoarthritis patients [14]. Firstly, it still can be argued whether CD34+/CD133+ cells truly represent circulating eEPCs, whose proliferative potential was investigated by us. Fadini et al. discussed the origin and phenotype of different EPC subtypes in a very detailed manner [17] and it can be doubted that the cells cultured in our study exclusively arise from CD34+/CD133+ cells. In addition, several own investigations showed that eEPC regeneration and numbers of circulating CD133+/KDR+ cells do not necessarily correlate in a positive manner [11, 13, 19].

Serum from SSc patients significantly reduced eEPC migration, a phenomenon which remained unaffected by Bosentan administration. After being incubated with serum from healthy Controls however cell migration was inhibited, potentially indicating that the cells were at least capable of interacting with the substance. Thus, serum from SSc patients may have reduced the cellular response to Bosentan which possibly indicates some degree of functional cell incompetence or Endothelial (Progenitor Cell) Dysfunction (ED) (37). In the past, anti-endothelin therapy has successfully been used in SSc with digital vasculopathy [38, 39] and endothelin has also been suggested to mechanistically contribute to the progression of SSc-associated fibrosis [40].

We would finally like to shortly discuss some limitations of the current study. In general, monitoring mesenchymal properties of circulating cells such as eEPCs allows to conclude about microvascular abnormalities only in an approximative manner. Secondly, the de-novo appearance of mesenchymal characteristics in eEPCs may potentially occur as bystander phenomenon within a systemic ‘pro-mesenchymal’ milieu. Thus, any eEPC ‘responsibility’ for aggravated skin and end-organ fibrosis may not be assumed. Finally, eEPCs represent only one of two EPC subpopulations. Investigations performed in recent years indicate that late EPCs (lEPCs) most likely are *true* progenitors of the endothelium whereas eEPCs support endothelial regeneration by indirect mechanisms [41]. Therefore, further studies should also address the role of the latter in SSc-associated fibrosis and microvasculopathy.

## Conclusions

In summary, the eEPC system, which represents an essential element in the endogenous vascular repair machinery is severely affected in SSc. The increased appearance of mesenchymal properties in eEPC may indicate that alterations of the cells contribute to or at least perpetuate the accumulation of connective tissue and the vascular malfunction.

## Abbreviations

ACR, American College of Rheumatology; aSMA, alpha Smooth Muscle Actin; CT, computed tomography; Dil-acLDL, Dil-labelled acetylated low density lipoprotein; dSSc, diffuse (generalized) systemic sclerosis; eEPCs, early endothelial progenitor cells; EULAR, European League Against Rheumatism; FSP-1, fibroblast specific protein-1; GPA, granulomatosis with polyangitis; lSSc, limited systemic sclerosis; PDGF, platelet derived growth factor; RA, rheumatoid arthritis; SSc, systemic sclerosis; TGF-b, transforming growth factor-beta; UE lectin, Ulex Europaeus lectin; VEGF, vascular endothelial growth factor

## Additional file

Additional file 1:
**Table S1.** Summarizes the baseline characteristics of all patients (see text for further details). (PDF 28 kb)
