# Supplementary material for: Early Endothelial Progenitor Cells (eEPCs) in systemic sclerosis (SSc) - dynamics of cellular regeneration and mesenchymal transdifferentiation
Source: BMC Musculoskelet Disord. 2016 Aug 12;17:339. doi: 10.1186/s12891-016-1197-2 (PMC4983068; doi:10.1186/s12891-016-1197-2)
Supplement: Additional file 1: — Table S1. Summarizes the baseline characteristics of all patients (see text for further details). (PDF 28 kb) [file 12891_2016_1197_MOESM1_ESM.pdf]

No.	type	gender	ANA	Scl70	Cenp-B	lungs	esophagus	joints	kidney	hypertension
**1**	limited	w	>1:640	0	pos			×	×	×
**2**	limited	w	>1:640	0	0			×		×
**3**	limited	m	nv	0	na					×
**4**	limited	m	>1:640	0	0					×
**5**	limited	w	>1:640	0	pos					
**6**	limited	w	>1:640	0	pos		×	×		×
**7**	limited	w	>1:640	0	pos					
**8**	limited	w	>1:640	0	0					
**9**	limited	w	>1:640	0	pos		×			×
**10**	limited	w	nv	0	0	×	×	×	×	×
**11**	limited	w	>1:640	pos	pos		×	×		×
**12**	limited	w	>1:640	0	pos			×		
**13**	limited	w	<1:640	0	pos					×
**14**	limited	w	>1:640	0	pos			×		
**15**	limited	w	<1:640	na	pos					×
**16**	limited	w	>1:640	0	pos		×	×		×
**17**	limited	w	>1:640	0	pos		×	×		×
**18**	limited	w	nv	na	na					
**19**	limited	w	>1:160	pos	pos					×
**20**	limited	w	>1:640	pos	pos			×		×
**21**	limited	w	>1:640	pos	pos					
**22**	diffuse	w	>1:20.000	na	0	×	×	×		
**23**	diffuse	w	<1:640	pos	0					
**24**	diffuse	m	<1:640	pos	0			×		×
**25**	diffuse	w	>1:640	0	0			×		
**26**	diffuse	m	>1:640	0	pos		×			×
**27**	diffuse	w	nv	pos	pos	×	×	×		
**28**	diffuse	w	>1:640	pos	0					
**29**	diffuse	w	>1:640	pos	0					×
